# Supplementary material for: Iterative improvement in the automatic modular design of robot swarms
Source: PeerJ Comput Sci. 2020 Dec 7;6:e322. doi: 10.7717/peerj-cs.322 (PMC7924708; doi:10.7717/peerj-cs.322)
Supplement: Supplemental Information 3 [file peerj-cs-06-322-s003.zip › argos3/doc/api/standalone/a00608_source.html]

ARGoS: plugins/simulator/physics\_engines/dynamics2d/dynamics2d\_gripping.cpp Source File


- Main Page
- Related Pages
- Namespaces
- Classes
- Files

- File List
- File Members

# plugins/simulator/physics\_engines/dynamics2d/dynamics2d\_gripping.cpp

Go to the documentation of this file.

```
00001 
00007 #include "dynamics2d_gripping.h"
00008 #include "dynamics2d_engine.h"
00009 
00010 #include <argos3/core/simulator/entity/embodied_entity.h>
00011 
00012 #include <algorithm>
00013 
00014 namespace argos {
00015 
00016    /****************************************/
00017    /****************************************/
00018 
00019    CDynamics2DGripper::CDynamics2DGripper(CDynamics2DEngine& c_engine,
00020                                           CGripperEquippedEntity& c_gripper_entity,
00021                                           cpShape* pt_gripper_shape) :
00022       m_cEngine(c_engine),
00023       m_cGripperEntity(c_gripper_entity),
00024       m_ptGripperShape(pt_gripper_shape),
00025       m_pcGrippee(NULL),
00026       m_tConstraint(NULL) {
00027       m_ptGripperShape->sensor = 1;
00028       m_ptGripperShape->collision_type = CDynamics2DEngine::SHAPE_GRIPPER;
00029       m_ptGripperShape->data = this;
00030       m_tAnchor = cpvzero;
00031    }
00032 
00033    /****************************************/
00034    /****************************************/
00035 
00036    CDynamics2DGripper::~CDynamics2DGripper() {
00037       Release();
00038    }
00039 
00040    /****************************************/
00041    /****************************************/
00042 
00043    void CDynamics2DGripper::CalculateAnchor(cpArbiter* pt_arb) {
00044       /* Calculate the anchor point on the grippable body
00045          as the centroid of the contact points */
00046       m_tAnchor = cpvzero;
00047       for(SInt32 i = 0; i < cpArbiterGetCount(pt_arb); ++i) {
00048          m_tAnchor = cpvadd(m_tAnchor, cpArbiterGetPoint(pt_arb, i));
00049       }
00050       m_tAnchor = cpvmult(m_tAnchor, 1.0f / cpArbiterGetCount(pt_arb));
00051    }
00052 
00053    /****************************************/
00054    /****************************************/
00055    
00056    void CDynamics2DGripper::Grip(CDynamics2DGrippable* pc_grippee) {
00057       m_tConstraint =
00058          cpSpaceAddConstraint(m_cEngine.GetPhysicsSpace(),
00059                               cpPivotJointNew(
00060                                  m_ptGripperShape->body,
00061                                  pc_grippee->GetShape()->body,
00062                                  m_tAnchor));
00063       m_tConstraint->maxBias = 0.95f;     // Correct overlap
00064       m_tConstraint->maxForce = 10000.0f; // Max correction speed
00065       m_cGripperEntity.SetGrippedEntity(pc_grippee->GetEmbodiedEntity());
00066       m_pcGrippee = pc_grippee;
00067       m_pcGrippee->Attach(*this);
00068    }
00069 
00070    /****************************************/
00071    /****************************************/
00072 
00073    void CDynamics2DGripper::Release() {
00074       if(IsGripping()) {
00075          cpSpaceRemoveConstraint(m_cEngine.GetPhysicsSpace(), m_tConstraint);
00076          cpConstraintFree(m_tConstraint);
00077          m_tConstraint = NULL;
00078          m_cGripperEntity.ClearGrippedEntity();
00079          m_pcGrippee->Remove(*this);
00080          m_pcGrippee = NULL;
00081       }
00082    }
00083 
00084    /****************************************/
00085    /****************************************/
00086 
00087    CDynamics2DGrippable::CDynamics2DGrippable(CEmbodiedEntity& c_entity,
00088                                               cpShape* pt_shape) :
00089       m_cEmbodiedEntity(c_entity),
00090       m_ptShape(pt_shape) {
00091       m_ptShape->collision_type = CDynamics2DEngine::SHAPE_GRIPPABLE;
00092       m_ptShape->data = this;
00093    }
00094 
00095    /****************************************/
00096    /****************************************/
00097 
00098    CDynamics2DGrippable::~CDynamics2DGrippable() {
00099       ReleaseAll();
00100    }
00101 
00102    /****************************************/
00103    /****************************************/
00104 
00105    void CDynamics2DGrippable::Attach(CDynamics2DGripper& c_gripper) {
00106       m_listGrippers.push_back(&c_gripper);
00107    }
00108 
00109    /****************************************/
00110    /****************************************/
00111 
00112    void CDynamics2DGrippable::Remove(CDynamics2DGripper& c_gripper) {
00113       CDynamics2DGripper::TList::iterator it =
00114          std::find(m_listGrippers.begin(), m_listGrippers.end(), &c_gripper);
00115       if(it != m_listGrippers.end()) {
00116          m_listGrippers.erase(it);
00117       }
00118    }
00119 
00120    /****************************************/
00121    /****************************************/
00122 
00123    void CDynamics2DGrippable::Release(CDynamics2DGripper& c_gripper) {
00124       CDynamics2DGripper::TList::iterator it =
00125          std::find(m_listGrippers.begin(), m_listGrippers.end(), &c_gripper);
00126       if(it != m_listGrippers.end()) {
00127          (*it)->Release();
00128       }
00129    }
00130 
00131    /****************************************/
00132    /****************************************/
00133 
00134    void CDynamics2DGrippable::ReleaseAll() {
00135       while(!m_listGrippers.empty()) {
00136          m_listGrippers.back()->Release();
00137       }
00138    }
00139 
00140    /****************************************/
00141    /****************************************/
00142 
00143    int BeginCollisionBetweenGripperAndGrippable(cpArbiter* pt_arb,
00144                                                 cpSpace* pt_space,
00145                                                 void* p_data) {
00146       /* Get the shapes involved */
00147       CP_ARBITER_GET_SHAPES(pt_arb, ptGripperShape, ptGrippableShape);
00148       /* Get a reference to the gripper data */
00149       CDynamics2DGripper* pcGripper = reinterpret_cast<CDynamics2DGripper*>(ptGripperShape->data);
00150       /* Get a reference to the grippable entity */
00151       CDynamics2DGrippable* pcGrippable = reinterpret_cast<CDynamics2DGrippable*>(ptGrippableShape->data);
00152       /* If the entities match, ignore the collision forever */
00153       return (&(pcGripper->GetGripperEntity().GetParent()) != &(pcGrippable->GetEmbodiedEntity().GetParent()));
00154    }
00155 
00156    /****************************************/
00157    /****************************************/
00158 
00159    int ManageCollisionBetweenGripperAndGrippable(cpArbiter* pt_arb,
00160                                                  cpSpace* pt_space,
00161                                                  void* p_data) {
00162       /* Get the shapes involved */
00163       CP_ARBITER_GET_SHAPES(pt_arb, ptGripperShape, ptGrippableShape);
00164       /* Get a reference to the gripper data */
00165       CDynamics2DGripper* pcGripper = reinterpret_cast<CDynamics2DGripper*>(ptGripperShape->data);
00166       /*
00167        * When to process gripping:
00168        * 1. when the robot was gripping and it just unlocked the gripper
00169        * 2. when the robot was not gripping and it just locked the gripper
00170        *    in this case, also precalculate the anchor point
00171        * Otherwise ignore it
00172        */
00173       if(pcGripper->IsGripping() && !pcGripper->IsLocked()) {
00174          /* Instruct the engine to remove the constraint in a post-step callback */
00175          cpSpaceAddPostStepCallback(
00176             pt_space,
00177             RemoveConstraintBetweenGripperAndGrippable,
00178             pcGripper,
00179             ptGrippableShape->data);
00180          return false;
00181       }
00182       if(!pcGripper->IsGripping() && pcGripper->IsLocked()) {
00183          pcGripper->CalculateAnchor(pt_arb);
00184          /* Instruct the engine to add the constraint in a post-step callback */
00185          cpSpaceAddPostStepCallback(
00186             pt_space,
00187             AddConstraintBetweenGripperAndGrippable,
00188             pcGripper,
00189             reinterpret_cast<CDynamics2DGrippable*>(ptGrippableShape->data));
00190          return false;
00191       }
00192       /* Always return false, anyway the gripper is a sensor shape */
00193       return false;
00194    }
00195    
00196    /****************************************/
00197    /****************************************/
00198 
00199    void AddConstraintBetweenGripperAndGrippable(cpSpace* pt_space,
00200                                                 void* p_obj,
00201                                                 void* p_data) {
00202       /* Get the objects involved */
00203       CDynamics2DGripper*   pcGripper   = reinterpret_cast<CDynamics2DGripper*>  (p_obj);
00204       CDynamics2DGrippable* pcGrippable = reinterpret_cast<CDynamics2DGrippable*>(p_data);
00205       /* Connect the objects */
00206       pcGripper->Grip(pcGrippable);
00207    }
00208    
00209    /****************************************/
00210    /****************************************/
00211 
00212    void RemoveConstraintBetweenGripperAndGrippable(cpSpace* pt_space,
00213                                                    void* p_obj,
00214                                                    void* p_data) {
00215       /* Get the gripper objects */
00216       CDynamics2DGripper* pcGripper   = reinterpret_cast<CDynamics2DGripper*>  (p_obj);
00217       /* Disconnect the objects */
00218       pcGripper->Release();
00219    }
00220    
00221    /****************************************/
00222    /****************************************/
00223 
00224 }
```

---

Generated on 10 Jul 2018 for ARGoS by 
 1.6.1
